# Study on Safe Filler Injection Techniques Using Cutting‐Edge Ultrasound Technology

**DOI:** 10.1111/jocd.71046

**Published:** 2026-07-08

**Authors:** Won Lee, Benjamin Ascher, Kyu‐Ho Yi, Ji‐Soo Kim, Audrey Melin, Seong Hwan Kim

**Affiliations:** ^1^ Yonsei E1 Plastic Surgery Clinic Anyang South Korea; ^2^ Think‐In Tech Paris France; ^3^ You & I Clinic (Mokdong) Seoul South Korea; ^4^ Dr Youth Clinic Seoul South Korea; ^5^ Iena21 Paris France; ^6^ Kangnam Sacred Heart Hospital, Hallym University College of Medicine Seoul Republic of Korea

**Keywords:** Doppler ultrasound, finger‐held ultrasound, hyaluronic acid filler, nasolabial fold, real‐time imaging

## Abstract

**Background:**

Recently, the development of an ultrasound device connected to a finger probe has enabled real‐time ultrasound‐guided filler injection. We aimed to evaluate the feasibility, clinical applicability, and patient satisfaction associated with the use of a novel real‐time finger‐held Doppler ultrasound system as a guide for hyaluronic acid filler injection.

**Methods:**

The SIBUS‐IN probe was attached to the fourth finger of the left hand, and hyaluronic acid filler was injected with the right hand.

**Results:**

A total of 33 patients underwent treatment for nasolabial folds, with an average injection of 0.98 mL (0.5–2) of Lorient No. 4 hyaluronic acid filler on the left side and 0.95 mL (0.5–1.5) of the same hyaluronic acid filler on the right side. Two weeks posttreatment, 22 out of 33 patients responded “very satisfied,” while 11 patients responded “satisfied.” All patients reported satisfactory results, with a mean satisfaction score of 2.67. No vascular complications were reported.

**Conclusions:**

The finger‐held Doppler ultrasound system (SIBUS‐IN) enabled real‐time visualization of vascular structures during hyaluronic acid filler injection and was feasible for clinical use in nasolabial fold correction. In this limited observational cohort, the technique was well tolerated and not associated with immediate adverse events. However, the present study was not designed to demonstrate safety superiority or complication prevention; controlled comparative studies are required to determine whether this approach offers measurable advantages in safety or outcomes over conventional injection techniques.

**Level of Evidence Statement:**

Level III.

## 1 Introduction

Hyaluronic acid filler injections are commonly performed minimally invasive aesthetic procedures [1]. However, these procedures may be associated with severe vascular adverse events, such as skin necrosis and ocular complications [2]. These issues are related to blood vessels, particularly facial arteries. Therefore, Doppler ultrasound procedures that allow preinjection assessment of various arteries may serve as useful adjuncts for vascular visualization and procedural planning [3].

Various danger zones on the face require anatomical knowledge, especially during hyaluronic acid filler injections [4], with the most dangerous areas being the glabellar area and nose [5]. The supratrochlear artery (glabellar region) [6], dorsal nasal artery (nasal region) [7], facial artery (nasolabial fold area) [8], and frontal branch of the superficial temporal artery (temporal region) [9] are all areas that should be identified via Doppler ultrasound before injection.

Traditional ultrasound procedures have primarily been used for vascular mapping before filler procedures [3], and real‐time ultrasound‐guided injections have been nearly impossible. Even when feasible, a practitioner is required to hold the ultrasound probe while the physician administers the hyaluronic acid filler.

Recently, the development of an ultrasound device that connects to a finger probe has made real‐time ultrasound‐guided filler injection technically possible. The present study evaluated the feasibility, clinical applicability, and patient satisfaction associated with the use of a novel real‐time finger‐held Doppler ultrasound system as a guide for hyaluronic acid filler injections. This study presents the use of a finger probe for ultrasound procedures in the facial area.

## 2 Materials and Methods

The SIBUS‐IN (Thinkin Tech SAS, Paris, France) ultrasound device was used in the present study. Ultrasound was activated using a high‐resolution 15‐MHz probe capable of imaging to a depth of approximately 1.5 cm–3 cm, and the settings including color imaging were adjusted to enhance vascular and tissue visualization.

A retrospective chart review was conducted on 33 patients, and injections were administered in the nasolabial fold. Injections were performed using Lorient No. 4 hyaluronic acid filler (Joonghun Pharmaceutical, Seoul, Republic of Korea). Lorient No. 4 hyaluronic acid filler exhibits moderate G', as demonstrated in previous literature on rheology (Table 1) [10].

**TABLE 1:** Various hyaluronic acid fillers tested.

Product	G' (Pa)	G" (Pa)	Complex viscosity (μ)	Tan delta	Cohesiveness (N)
Lorient No. 2	203	41	1 673 007	0.20	0.4401
Lorient No. 4	338	95	2 795 776	0.28	0.4237
Lorient No. 6	413	121	3 423 232	0.29	0.4454

*Note:* (Frequency 0.02 Hz) MCR 301 rheometer (Anton Paar Co., Graz, Austria).

**FIGURE 1:**
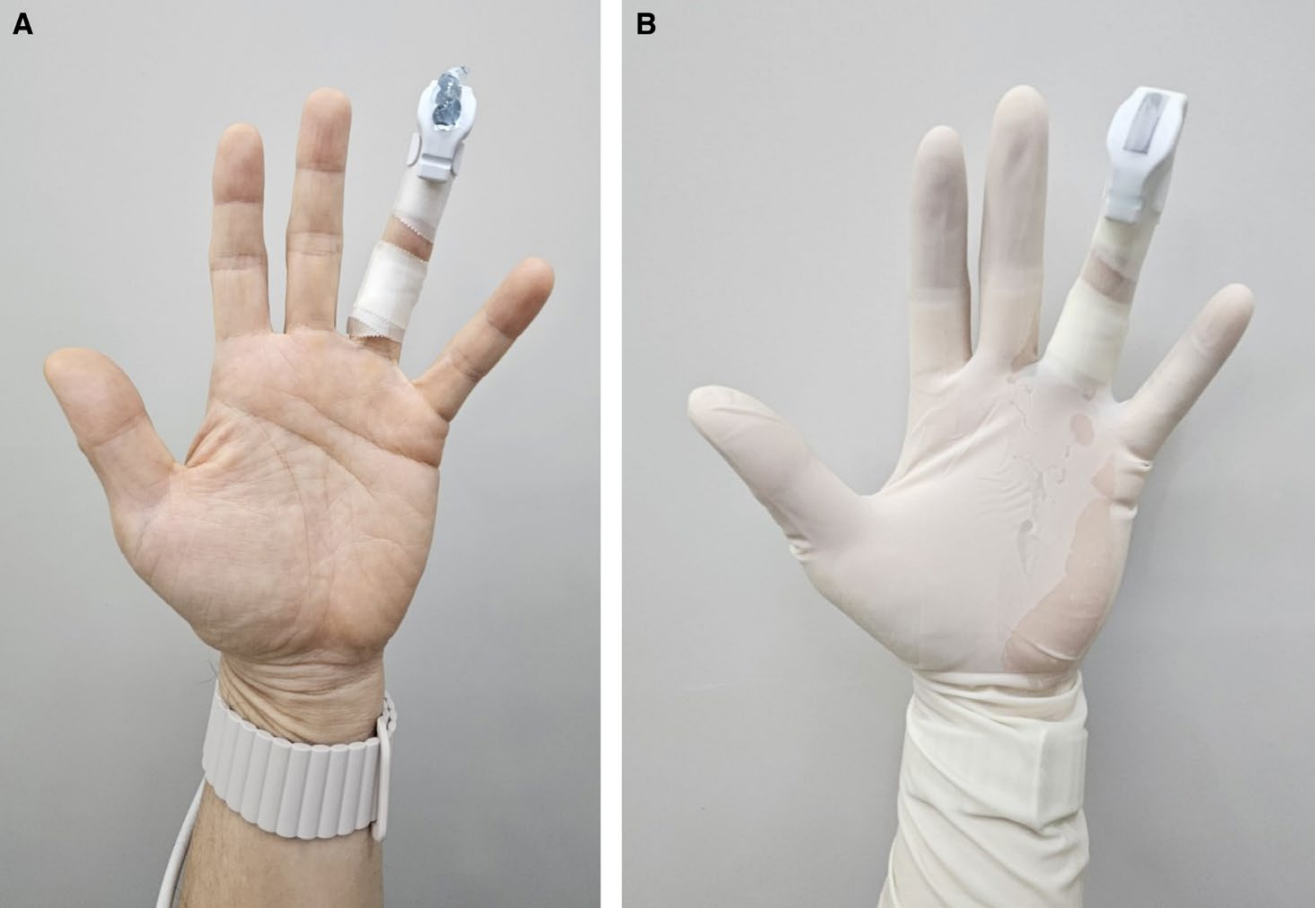
Method of wearing the ultrasound probe.

The ultrasound probe was attached to the fourth finger of the left hand of the doctor (Figure 1a). The length of the probe is approximately 1 cm. After applying ultrasound gel, sterile gloves were worn (Figure 1b). The face of the patient was completely disinfected with chlorhexidine. An antibiotic ointment was applied over the glove to ensure sterile conditions.

**FIGURE 2:**
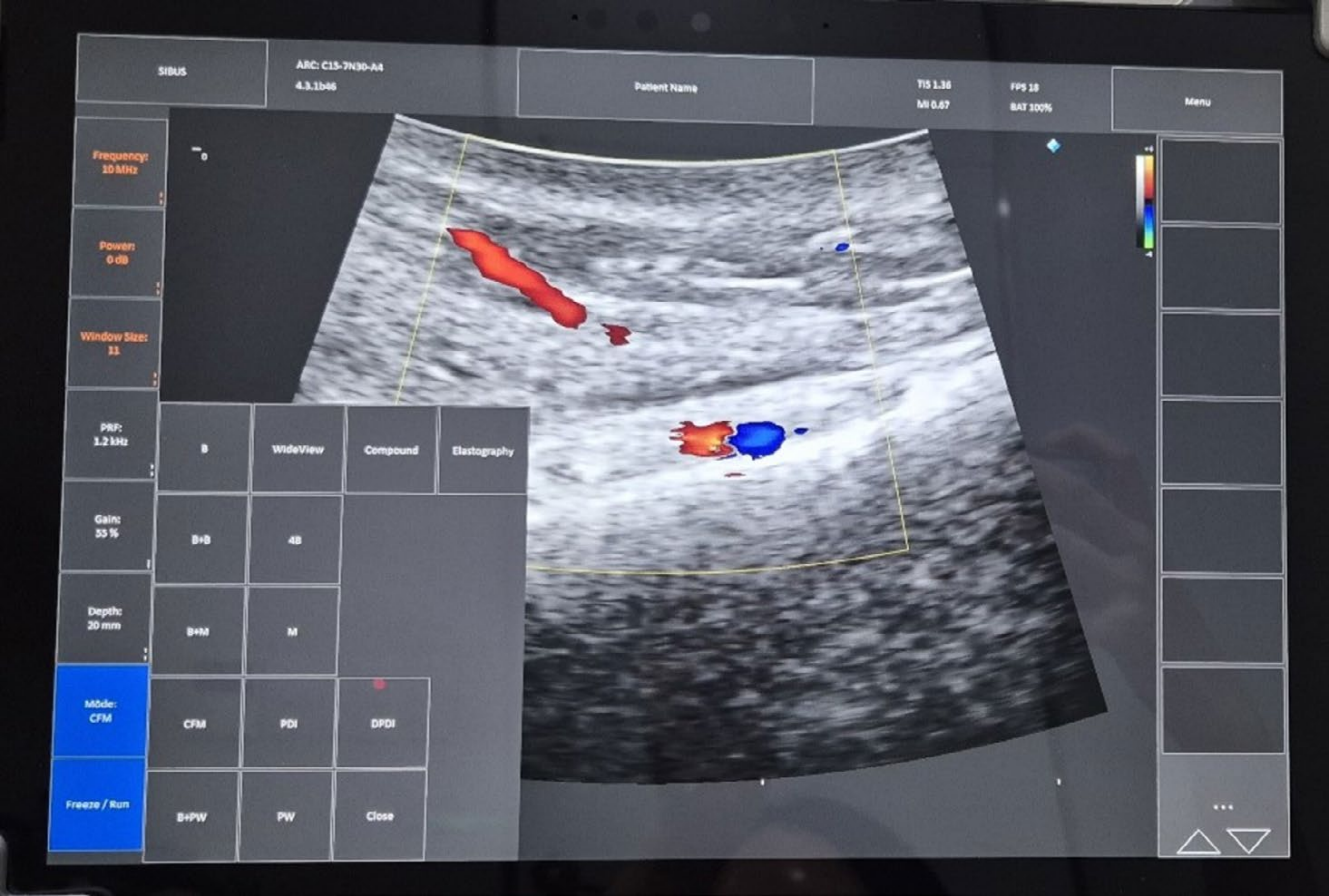
Doppler ultrasound setting. High‐resolution 15 MHz was applied.

The optimal settings (frequency, power, window size, gain, and Doppler mode) were determined after positioning the probe (fourth finger of the hand of the doctor) on the face of the patient (Figure 2). As the primary goal was to detect the artery while injecting the filler, the device was set to the Doppler mode instead of the B mode.

**FIGURE 3:**
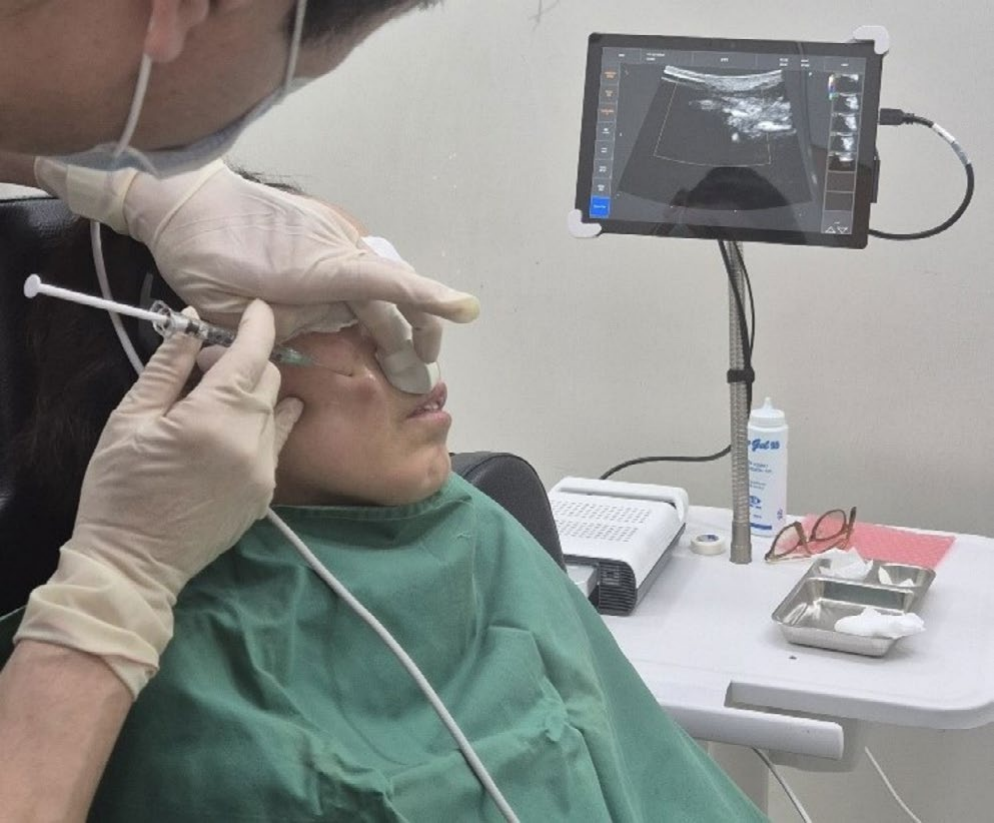
Ultrasound‐guided hyaluronic acid filler injection. Detection of the cannula in the monitor.

A puncture site was made at the intersection of a virtual vertical line from the lateral canthus and a virtual horizontal line from the nasal alar area. A puncture was made lateral to the nasolabial fold using a 19G needle, followed by filler injection with a 21G cannula (Figure 3).

**FIGURE 4:**
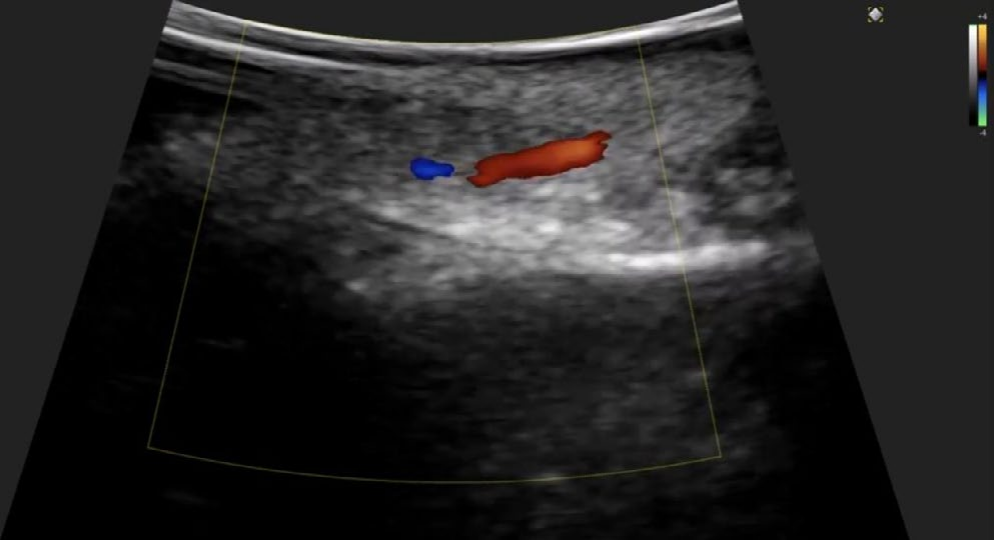
Detection of the facial artery located in the subcutaneous fat layer above the nasolabial fold.

The ultrasound probe detected the facial artery at the nasolabial fold area and initially confirmed the facial artery pathway (Figure 4; Video S1).

The cannula was primarily visualized in the in‐plane ultrasound mode, allowing horizontal alignment with vascular structures and facilitating positioning to avoid adjacent vessels during injection.

**FIGURE 5:**
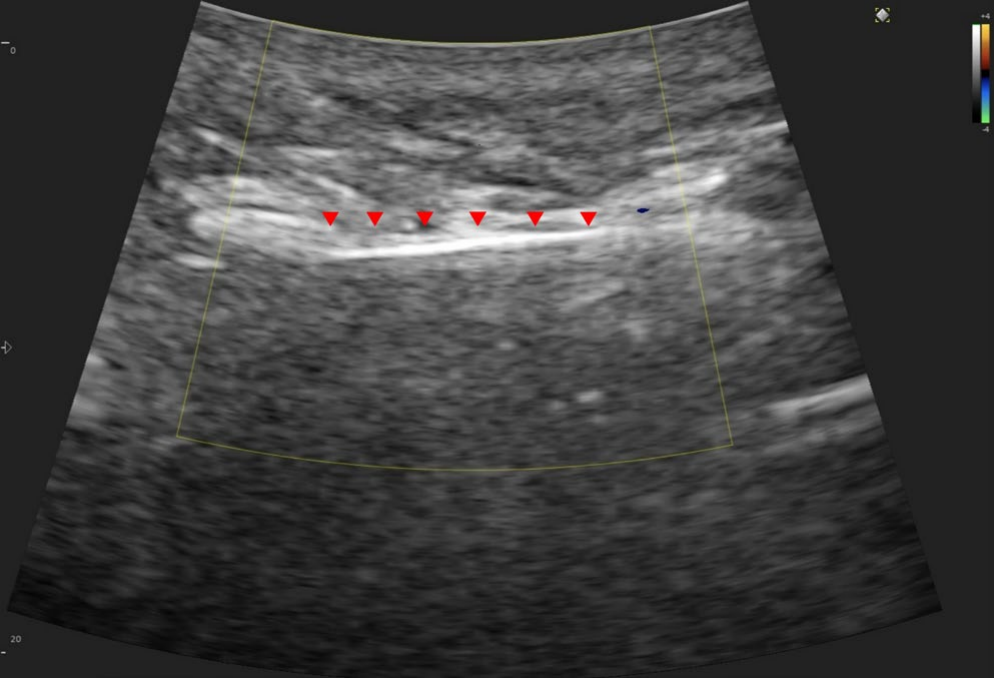
In‐plane mode ultrasound image. The red arrowhead indicates the cannula.

After avoiding the blood vessels, the cannula tip was approached and confirmed using ultrasound (Figure 5). When the cannula was positioned away from the vessel, the hyaluronic acid filler was gradually injected (Video S2).

No adverse effects were observed. Photographs were taken before the procedure, and 2 weeks and 1 month after filler injection. Patient satisfaction was evaluated 2 weeks postoperatively. A four‐point questionnaire was administered, with the scoring as follows: 3 = “very satisfied,” 2 = “satisfied,” 1 = “barely satisfied or dissatisfied,” and 0 = “worse.” The Global Aesthetic Improvement Scale (GAIS) and the Wrinkle Severity Rating Scale (WSRS) were used for evaluation. GAIS scores were assessed by two board‐certified plastic surgeons. The GAIS was scored by comparing preoperative and 1‐month postoperative photographs as follows: 3 points (very much improved), 2 points (much improved), 1 point (improved), 0 points (no change), and −1 point (worse). Additionally, nasolabial fold severity was evaluated using the WSRS at the same time points (pretreatment and 1 month treatment). WSRS scores were as follows: 5 points (Extreme: extremely deep and long folds; 2 mm–4 mm visible v‐shaped fold when stretched; detrimental to appearance; and unlikely to have satisfactory correction with injectable implant alone), 4 points (Severe: very long and deep; prominent facial feature; and less than 2 mm visible fold when stretched), 3 points (Moderate: moderately deep fold; clear facial feature visible at normal appearance but not when stretched; and excellent correction expected), 2 points (Mild: shallow but visible fold with slight indentation; and minor facial feature), and 1 point (Absent: no visible fold; and continuous line).

This retrospective study was approved by the institutional review board (IRB number 2024‐09‐005). All procedures involving human participants were performed in accordance with the ethical standards established by the institutional and/or national research committee and with the 1964 Helsinki declaration and its later amendments or comparable ethical standards.

## 3 Results

From December 3, 2024, to February 28, 2025, 33 patients (28 women, 5 men, mean age of 51.5 years) underwent treatment for nasolabial folds. We injected an average of 0.98 mL (range: 0.5 mL–2 mL) of Lorient No. 4 hyaluronic acid filler on the left side and 0.95 mL (range: 0.5 mL–1.5 mL) on the right side. Two weeks posttreatment, 22 of 33 patients responded as being “very satisfied,” while 11 patients responded as being “satisfied.” All patients reported satisfactory results, with a mean satisfaction score of 2.67.

**FIGURE 6:**
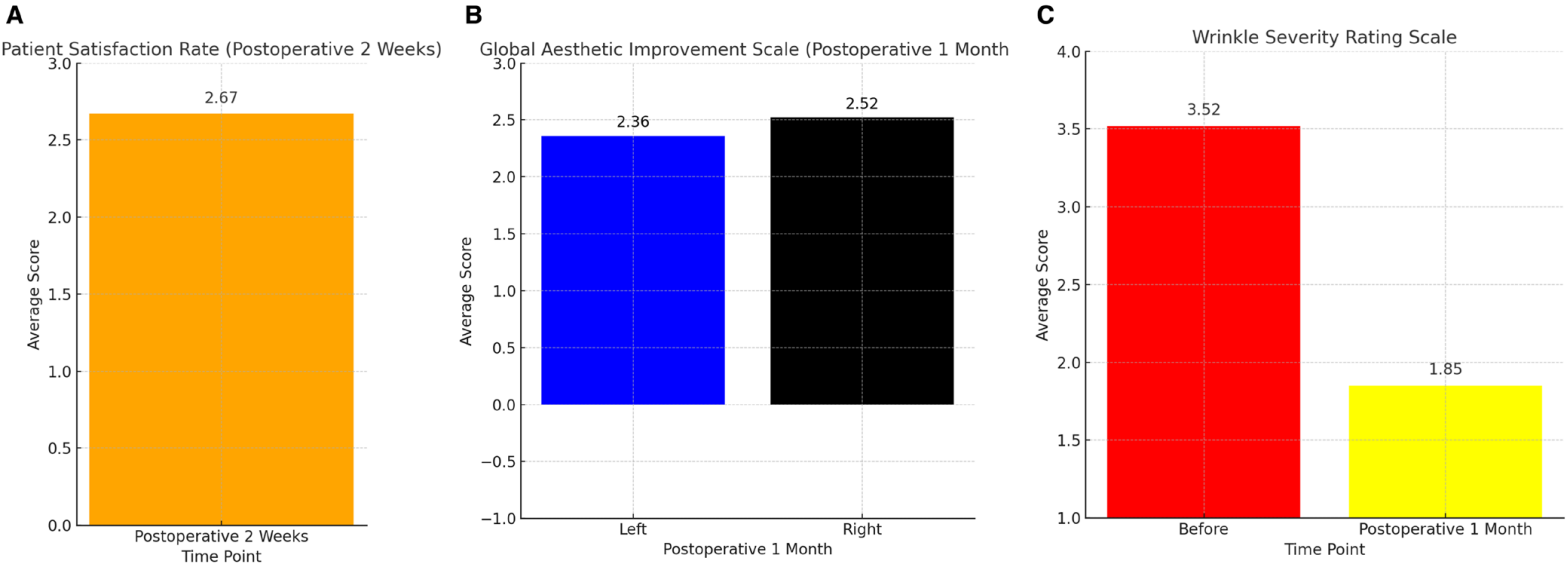
Patients' results (A) Patient satisfaction rate postoperative 2 weeks, (B) GAIS, and (C) WSRS.

Two board‐certified plastic surgeons evaluated the photographs using the GAIS and the WSRS. For the GAIS (physician‐assessed), at 1 month posttreatment, the average score for the left side of the face ranged from 3 to 1, with a mean of 2.36. The right side ranged from 3 to 2, with a mean of 2.52. For the WSRS, pretreatment scores ranged from 4 to 2, with a mean of 3.52. At 1 month posttreatment, scores ranged from 3 to 1, with a mean of 1.85 (Figure 6).

No immediate adverse events, including vascular complications, were observed during the follow‐up period, and no additional injections were required. One patient complained of asymmetry immediately after injection but showed symmetry after the swelling subsided.

In this study, patients were monitored for approximately 1 month for the occurrence of complications following the procedure. Patient satisfaction was assessed at 2 weeks using the GAIS, and clinical improvement was evaluated at 1 month using the WSRS.

The relatively short follow‐up period was intentionally selected because the primary focus of this investigation was to observe the feasibility and clinical applicability of real‐time ultrasound visualization during filler injection, rather than to evaluate the long‐term aesthetic effects or longevity of the filler. Therefore, assessments at 2 weeks and 1 month were considered appropriate for the objectives of this study.

## 4 Discussion

Vascular complications remain among the most serious adverse events associated with filler procedures [2], primarily resulting from arterial compression or occlusion, which may lead to skin necrosis or ocular complications. Consequently, various preventive strategies have been proposed, many of which are based on expert consensus [11]. Among these approaches, Doppler ultrasound has been increasingly utilized to visualize vascular structures at planned injection sites [3].

The face contains various blood vessels, which can exhibit structural and other variations. By using Doppler ultrasound, the course and depth of these vessels can be assessed [12]. Performing Doppler ultrasound in areas referred to as “danger zones” may be helpful for vascular assessment and injection planning. The glabellar region is where the supratrochlear artery runs, and injecting filler in this area significantly increases the risk of ocular complications [13]. This area can be confirmed via ultrasound, and in case the vessel is located in the glabellar wrinkle, avoiding filler injection is advisable [6]. The forehead is also a common site for filler injections. In this case, confirming the course of the supraorbital artery before injection is recommended [14]. The position of the deep temporal artery can also be confirmed in the temporal region [15]. The facial artery, which may exhibit various layers and detour branch variations, traverses the nasolabial fold area [8]. Recent studies have demonstrated that the depth of the facial artery varies widely [16]. Therefore, real‐time ultrasound visualization may be useful for identifying vascular structures during filler injection. Among the various facial regions, this study focused on the area where the facial artery can be most clearly visualized, selecting a site that allows the most practical and convenient use of the finger‐held probe during filler injection.

Several publications have reported the utility of Doppler ultrasound for vascular visualization during filler procedures, and many practitioners consider ultrasound guidance a potentially useful adjunct to conventional techniques [3]. However, most existing applications have focused on preinjection vascular mapping rather than on real‐time monitoring during filler injection.

Real‐time ultrasound guidance during filler injection typically requires two operators—one to hold the probe and another to inject. In the present study, the finger‐mounted ultrasound probe allowed simultaneous real‐time vessel visualization and hyaluronic acid filler injection by a single practitioner. While the SIBUS‐IN (Thinkin Tech SAS) protocol recommends attachment of the probe to the second finger, we found that positioning the probe on the fourth finger provided improved handling and procedural ergonomics during injection.

Although this study focused on nasolabial fold correction, the same real‐time visualization approach may be applicable to other facial injection sites where vascular structures are of concern.

Several limitations should be acknowledged. First, the study involved a relatively small cohort of 33 patients. Second, the retrospective, non‐comparative design without a control group precludes conclusions regarding superiority, improved safety, or reduction in vascular complications compared with conventional injection techniques or other ultrasound modalities. While prior literature supports the role of Doppler ultrasound in vascular visualization during filler procedures, the present study primarily demonstrates feasibility and clinical applicability rather than comparative effectiveness. Third, ultrasound‐guided techniques require dedicated training, and the smaller probe size used in the SIBUS‐IN system may result in reduced image clarity compared with conventional devices. However, several studies using finger‐held ultrasound systems have already been published and have discussed aspects of procedural safety [17, 18]. Nevertheless, further research is still required to more definitively evaluate clinical outcomes and safety benefits. Nevertheless, appropriate training may improve image interpretation, underscoring the importance of structured ultrasound education in aesthetic practice [19]. Further prospective, controlled, or split‐face studies will be necessary to validate potential clinical benefits and long‐term outcomes.

## 5 Conclusions

The finger‐held Doppler ultrasound system (SIBUS‐IN) enabled real‐time visualization of vascular structures during hyaluronic acid filler injection and was feasible for clinical use in nasolabial fold correction. In this limited observational cohort, the technique was well tolerated and not associated with immediate adverse events. While these findings suggest potential clinical utility, the present study does not demonstrate safety superiority or prevention of vascular complications. Controlled comparative studies are required to determine whether this approach offers measurable advantages in safety or outcomes over conventional injection techniques.

## Author Contributions

Conceptualization: W.L., B.A., K.‐H.Y., and J.‐S.K.; methodology: W.L.; software: W.L.; validation: W.L., K.‐H.Y., and J.‐S.K.; formal analysis: W.L.; investigation: W.L.; resources: W.L.; data curation: W.L.; writing – original draft preparation: W.L., B.A., and S.H.K.; writing – review and editing: W.L., B.A., A.M., and K.‐H.Y.; visualization: B.A., A.M., and J.‐S.K.; supervision: S.H.K.; project administration: S.H.K. All authors have read and agreed to the published version of the manuscript.

## Funding

This research was supported by Hallym University Medical Center Research Fund.

## Ethics Statement

This retrospective study was approved by the Institutional Review Board of Kangnam Sacred Heart Hospital (IRB number 2024‐09‐005). All procedures involving human participants were performed in accordance with the ethical standards established by the institutional and/or national research committee and with the 1964 Helsinki declaration and its later amendments or comparable ethical standards.

## Conflicts of Interest

Dr. Benjamin Ascher is affiliated with Think‐In Tech, Paris, France, the developer of the SIBUS‐IN ultrasound device discussed in this manuscript, and has contributed to the development of the product. This disclosure is provided to ensure transparency regarding potential conflicts of interest. All other authors declare no conflicts of interest.

## Supporting information


**Video S1:** Real‐time Doppler ultrasound visualization of the facial artery pathway in the nasolabial fold area using a finger‐held ultrasound probe.

**Video S2:** Real‐time ultrasound‐guided hyaluronic acid filler injection into the nasolabial fold after confirming the cannula tip position away from the facial artery.

## Data Availability

The data that support the findings of this study are available on request from the corresponding author. The data are not publicly available due to privacy or ethical restrictions.
